# Points for energy renovation (PointER): A point cloud dataset of a million buildings linked to energy features

**DOI:** 10.1038/s41597-023-02544-x

**Published:** 2023-09-20

**Authors:** Sebastian Krapf, Kevin Mayer, Martin Fischer

**Affiliations:** 1 Institute of Automotive Technology, Department of Mechanical Engineering, TUM School of Engineering and Design, Technical University of Munich, Boltzmannstr. 15, 85748 Garching b. München, Germany; 2 Department of Civil and Environmental Engineering, Stanford University, 473 Via Ortega, 94305 Stanford, USA

**Keywords:** Civil engineering, Scientific data, Attribution, Energy efficiency, Energy supply and demand

## Abstract

Rapid renovation of Europe’s inefficient buildings is required to reduce climate change. However, evaluating buildings at scale is challenging because every building is unique. In current practice, the energy performance of buildings is assessed during on-site visits, which are slow, costly, and local. This paper presents a building point cloud dataset that promotes a data-driven, large-scale understanding of the 3D representation of buildings and their energy characteristics. We generate building point clouds by intersecting building footprints with geo-referenced LiDAR data and link them with attributes from UK’s energy performance database via the Unique Property Reference Number (UPRN). To mimic England’s building stock’s features well, we select one million buildings from a range of rural and urban regions, of which half a million are linked to energy characteristics. Building point clouds in new regions can be generated with our published open-source code. The dataset enables novel research in building energy modeling and can be easily expanded to other research fields by adding building features via the UPRN or geo-location.

## Background & Summary

Buildings are responsible for 40% of the European Union’s energy consumption and 36% of its greenhouse gas emissions^1^. In the residential sector, space heating, cooling, and hot water supply constitute up to 80% of citizens’ energy consumption. At the same time, more than 75% of EU buildings are inefficient^2^, and the share of the building stock that undergoes major renovation is low, ranging from less than 0.4% to 1.2% across EU member states^1^. Therefore, renovating buildings is a key initiative of the European Green Deal^3^. To this end, the European Commission published the “A Renovation Wave for Europe” strategy that aims to tackle inefficient buildings and to double annual energy renovation rates^4^.

In practice, Energy Performance Certificates (EPC) are one of the central instruments to contribute to these goals by providing transparency of the existing building stock’s energy efficiency. It is mandatory to issue an EPC for buildings up for sale or rent and to display them in advertisements^5^. In Europe, the United Kingdom has the largest number of registered EPCs – more than 20 Million^5^ – with around 60% of coverage in England and Wales in 2022^6^. EPCs are created for existing buildings by accredited energy assessors during on-site visits^7^, which makes the process slow, costly, and local. To achieve the aggressive renovation targets, methods are needed that generate relevant insights to buildings based on widely available data sources.

To this end, there is increasing research interest, in particular in the field of urban building energy modeling (UBEM)^8–10^. UBEM refers to the bottom-up building energy modeling and analysis on a city-scale. One aim of UBEM is to identify inefficient buildings automatically on large scale with continuous coverage. However, Ali *et al*. point out that necessary input data is often unavailable for an entire city or a district^9^. Moreover, the prevalent physics-based or engineering approach in UBEM is currently inadequate for large-scale building-level analysis because it requires highly detailed input data^9^.

As an alternative to physics-based modeling, some researchers propose data-driven approaches which aim at determining energy characteristics with fewer input data^11–16^. Publications mention different compositions of input features such as occupancy, year of construction, insulation of building envelope, or surface-to-volume ratio. However, these features are usually only available for a specific region or a subset of buildings in an area of interest.

Consequently, researchers turn to extracting relevant features, or proxies that indirectly indicate features, from remotely sensed data, which is available on a larger scale^16–21^. For example, building footprints^16^ and roof shapes^18^ can be gathered from aerial images. Street view images include information about the window-to-wall ratio or the number of floors^19,21^. Airborne LiDAR contains information about a building’s height^15^ and can be further processed into Digital Surface Models (DSM)^19,20^ or 3D models^22^ to reflect a building’s envelope and volume. Implicitly, LiDAR also contains information that is relevant for building energy modeling. For example, Tooke *et al*. predicted building age based on LiDAR^17^, and Castagno and Atkins improved roof shape classification with LiDAR^18^.

In summary, combining widely available remotely sensed data sources with data-driven algorithms to estimate energy efficiency can provide building insights fast and at scale^21^. LiDAR is a promising data source as it includes features linked to building height, envelope geometry, roof superstructures, and surface-to-volume ratio. In addition, age, building type, or architectural style can be inferred from a building’s point cloud representation.

To support and accelerate research in this field, this paper provides a large-scale dataset of building point clouds coupled with energy characteristics. The major contributions are two-fold:

1. The Points for Energy Renovation (PointER) dataset^23^ comprises more than 1 million building point clouds and covers 16 diverse authority districts in England. When available, building point clouds are linked to data from UK’s energy performance database, leading to more than half a million complete sets of point clouds with energy characteristics. The dataset can be downloaded from 10.14459/2023mp1713501
2. Open source code with a detailed documentation to replicate the building point cloud generation process enables follow-up studies. The process is region-agnostic and can be applied anywhere where LiDAR data and building footprints are available. The code is available at https://github.com/kdmayer/PointER

## Methods

**Fig. 1:**
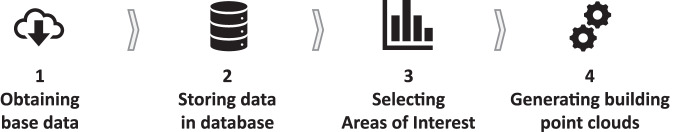
Data pipeline overview: generating a building point cloud dataset in four steps.

Figure 1 gives a high-level overview of the conducted steps to create the PointER dataset^23^.

### Obtaining base data

Multiple open datasets in the UK enabled the creation of the PointER building point cloud dataset, including LiDAR data and Europe’s largest collection of EPCs. In this study, we focused on England, as not all of the required data was available for the entire UK. To produce the dataset, we obtained seven base datasets which are summarized in Table 1. All datasets were provided under the Open Government License (OGL) except for the building footprints dataset.

**Table 1:** Overview of base data sources.

	Acronym	Dataset name	Years	License	Ref.
1	Point clouds	National LIDAR Programme Point Cloud	2017–2021	OGL	^24^
2	Verisk building footprints	UKBuildings edition 13 online version	2021	Personal License**	^26^
3	EPC	Energy Performance of Buildings Data: England and Wales	2008–2022	OGL*	^29^
4	UPRN	Ordnance Survey Open Unique Property Reference Number	2022	OGL	^30^
5	LAD	Local Authority Districts (Dec 2021) GB BFC	2021	OGL	^31^
6	RUC	2011 Rural Urban Classification lookup tables for all geographies	2011	OGL	^32^
7	OA	Output Areas (Dec 2011) Boundaries EW (BFC)	2011	OGL	^34^

*except for address information.

**we obtained a personal license from Verisk for the research project and the permission to publish the resulting point clouds. The original footprint data cannot be shared or published.

The first and major component was UK’s National LiDAR Programme Point Cloud data, collected between 2017 and 2021. The LiDAR data covers all of England with a resolution of 1 m and is available online^24^. The point data is categorized into the ASPRS Standard LiDAR Point Classes^25^, such as building, ground, low vegetation, water, etc. Hence, building point clouds could simply be extracted from the LiDAR data based on their classification. However, to match energy characteristics on the property level, we required a distinction between neighboring properties. In particular, the boundaries of properties could not be extracted from LiDAR points alone in the common case of townhouses. Therefore, our alternative approach was to crop points using building footprint data.

**Fig. 2:**
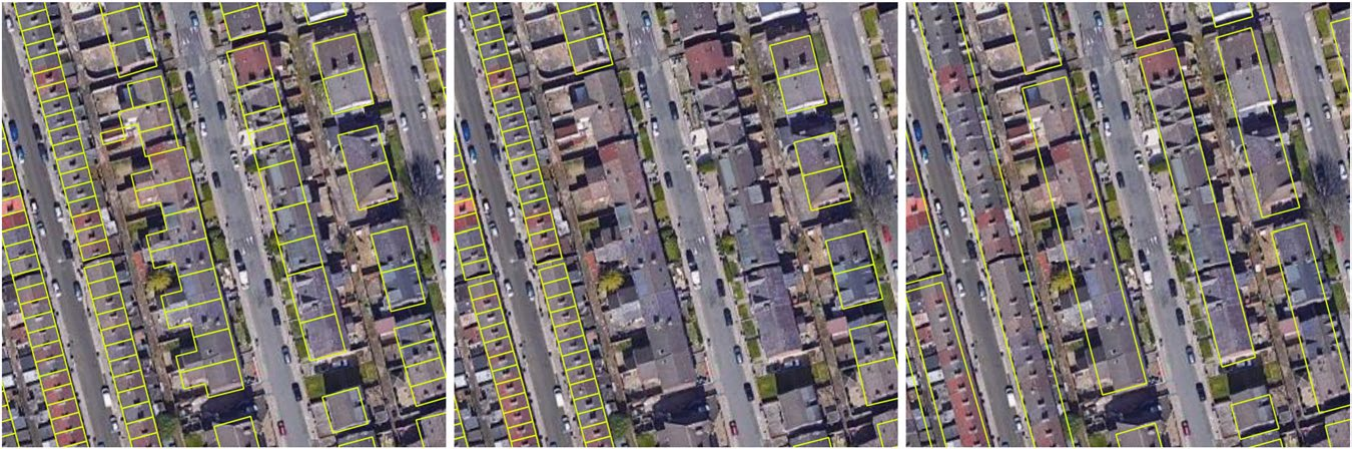
Building outline polygons of three available datasets visualized on aerial images. UKBuildings^26^ (left), OSM^27^ (center), and OS OpenMap^28^ (right) on Google Maps aerial image.

To this end, we required accurate building footprint data. We chose the UKBuildings edition 13 online version dataset by Verisk^26^ because it had the most accurate data based on our visual analysis of aerial images. Figure 2 displays UKBuildings’ polygons as well as building outlines from OpenStreetMaps (OSM)^27^ and Ordnance Survey’s OS OpenMap - Local^28^. As can be seen from the examples, a common downside of OSM was the missing footprint information. Likewise, the OS dataset was not suitable because it did not contain exact property lines between the buildings. While Verisk’s footprint data has a higher quality, the drawback is that we could not publish the footprint data due to the private license.

Our goal was to link the point clouds to energy performance certificate data^29^. The certificates are available for a subset of properties in England and Wales. There are currently around 25.60 million domestic EPCs and 1.11 non-domestic EPCs in the database^6^. The main label is an energy rating between “A” for most efficient and “G” for least efficient. However, the EPC database includes more detailed information, such as roof insulation efficiency or window efficiency, which were also added as energy attributes. The data is available under the OGL, except for the Ordnance Survey address data entries, which were therefore excluded from our dataset.

To link the EPC with the building point clouds, we used the Unique Property Reference Numbers (UPRN), which exist for most of the UK’s buildings^30^. EPC data is structured by local authority districts (LAD). Accordingly, we also downloaded LADs’ boundary shape files provided by the Office for National Statistics^31^ to limit the point cloud generation to geographic Areas of Interest (AOI).

Lastly, we aimed at generating a dataset of buildings that possess a similar composition as the entire English building stock. We used the 2011 Rural Urban Classification^32^. The classification distinguishes ten rural urban classes ranging from “rural hamlets and isolated dwellings in a sparse setting” to “urban major conurbation”. We assigned one of these classes to each of the footprints. This allowed us to select AOIs so that our dataset possessed the same rural urban distribution as entire England. The smallest available geographic entities are the Output Areas (OA). OAs entail an average resident population of approximately 300 people^33^. Therefore, we obtained the OA boundary shape files^34^ as the last data source.

### Storing data in a database

A key element of the dataset generation process was a postgres^35^ database to store the obtained data. We used the postGIS^36^ extension for geo-data and the pgpointcloud^37^ extension for the point cloud data, as well as gdal^38^ and pdal^39^ to insert the data into the database, respectively. The program itself was written in Python3. The program manipulates data and interacts with the postgres database. The code and utilized Python libraries can be found at https://github.com/kdmayer/PointER. The framework was set up in a Singularity^40^ container. Most of the data was inserted into the database before running the point cloud generation script. However, point cloud and EPC data were imported periodically during the runtime for one AOI only. This reduced the amount of data in the database and speeded up the cropping process.

### Selecting areas of interest

**Fig. 3:**
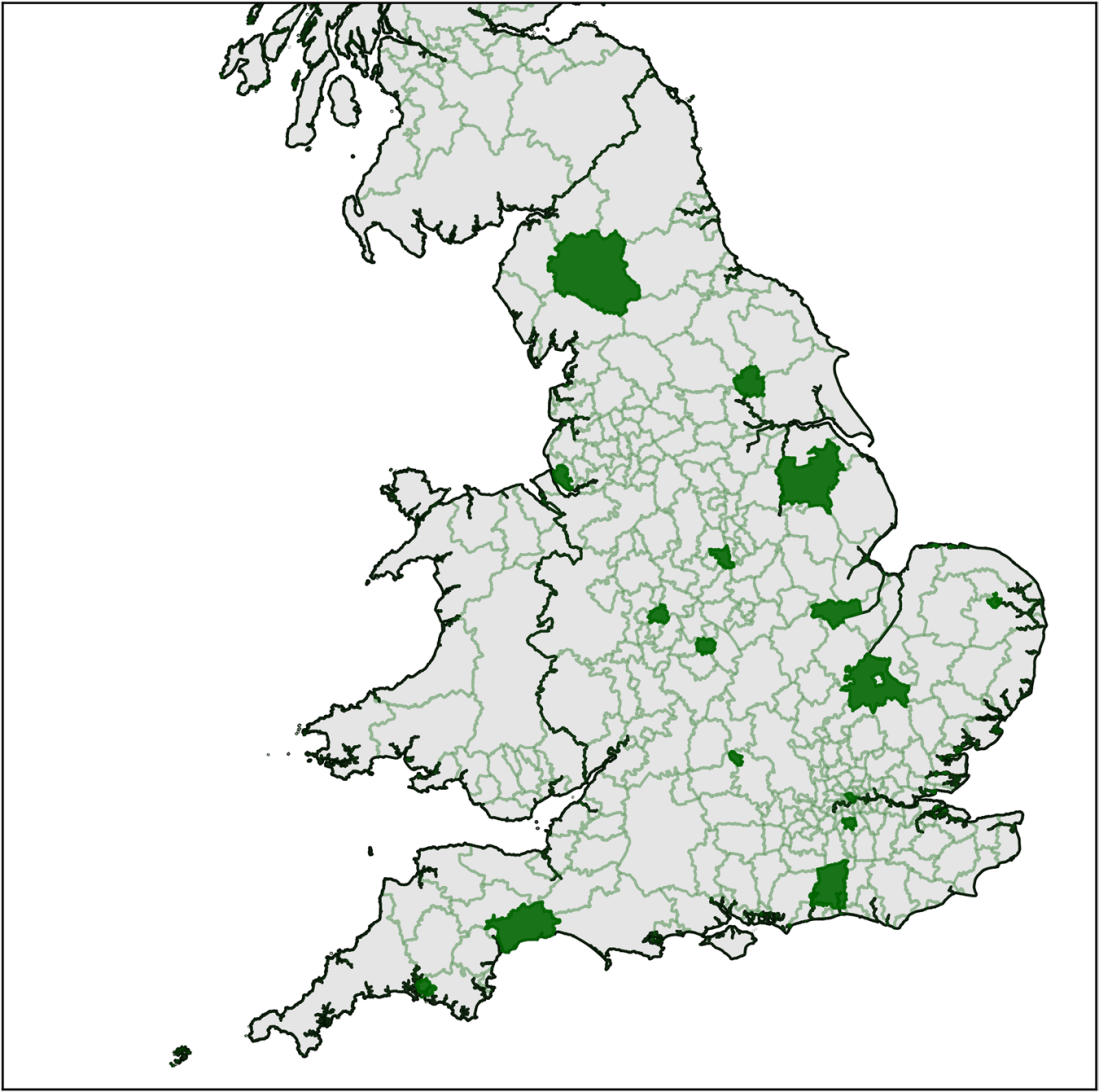
Map of UK’s Local Authority Districts (LADs) and the 16 LADs selected for our dataset highlighted in green.

To generate point clouds for a subset of England’s building stock, we chose a geographically diverse set of LADs. We first selected Coventry, Westminster, Oxford, and Peterborough, similarly to Mayer *et al*.^21^. Furthermore, we used the RUC for small geographies to calculate England’s RUC distribution and all LAD’s distribution. We chose LADs with the goal to reach a RUC distribution similar to England in our dataset. To account for the geographic diversity of buildings in England, we selected LADs from across the country including coastal and interior regions. Figure 3 visualizes the geographic distribution of selected LADs. Furthermore, columns one and two of Table 2 give and overview of selected LADs, sorted by district code.

**Table 2:** Overview of number of footprints with point cloud, UPRN and EPC data in the dataset.

LAD Code	Name	# footprints	# FP with point clouds	# FP with UPRN	# FP with UPRN & EPC	FP with full info
E06000014	*York*	85.551	89%	84%	47%	44%
E06000026	*Plymouth*	154.151	62%	60%	38%	33%
E06000031	*Peterborough*	80.532	80%	84%	56%	46%
E07000012	*South Cambridgeshire*	69.179	83%	67%	41%	38%
E07000030	*Eden*	35.264	88%	58%	32%	29%
E07000036	*Erewash*	72.133	65%	62%	35%	29%
E07000040	*East Devon*	76.142	83%	68%	41%	36%
E07000142	*West Lindsey*	51.758	83%	66%	39%	36%
E07000148	*Norwich*	66.050	75%	73%	47%	42%
E07000178	*Oxford*	49.882	69%	85%	54%	37%
E07000227	*Horsham*	63.711	83%	68%	39%	36%
E08000012	*Liverpool*	202.656	76%	88%	53%	42%
E08000026	*Coventry*	138.458	64%	86%	51%	33%
E08000030	*Walsall*	116.036	81%	84%	47%	41%
E09000029	*Sutton*	96.167	65%	57%	30%	28%
E09000033	*Westminster*	27.371	96%	84%	54%	54%
	***Absolute sum***	1.385.041	**1.040.425**	1.038.406	619.554	**518.992**

Our data includes other properties besides rural urban classification, such as building age, height, size and energy related features. In our dataset, these properties’ distribution should ideally be similar to the distribution of the entire English building stock. While we did not take this data into account for the selection of regions to reduce complexity, we analyzed these properties in retrospect and included the result in the chapter Technical Validation.

### Generating building point clouds

**Fig. 4:**
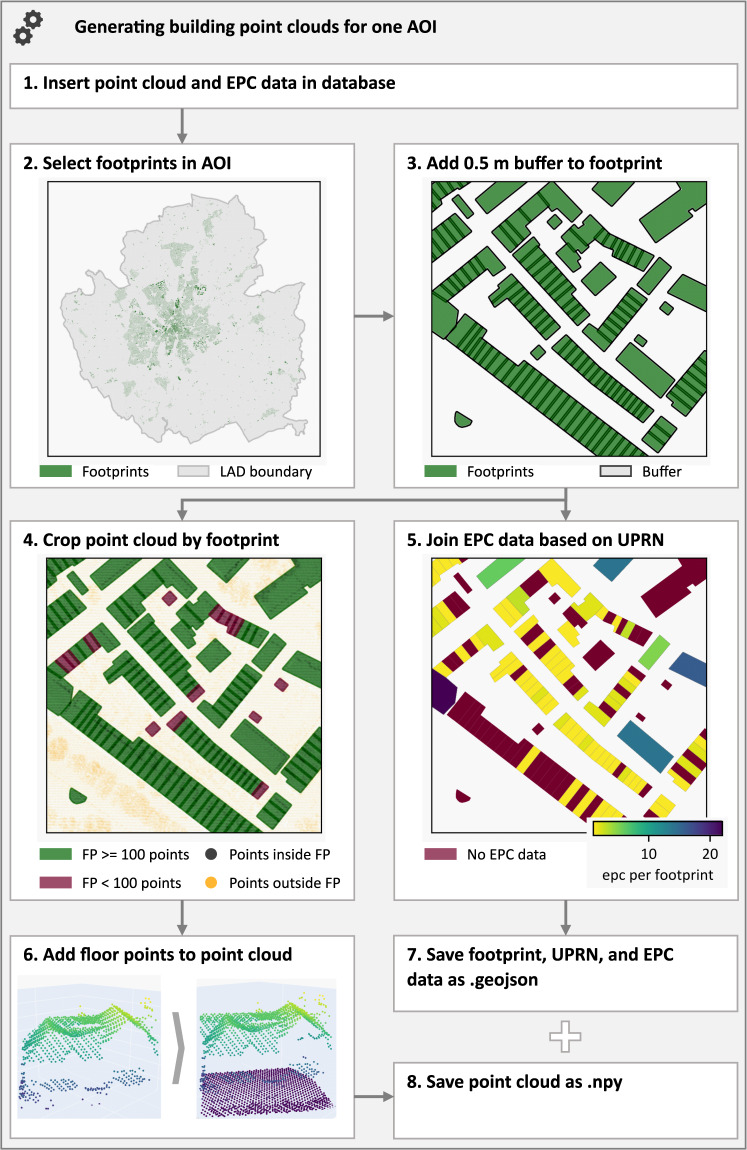
Overview and visualization of the program’s steps for generating building point clouds and their energy characteristics for one AOI.

Building point clouds were generated for one AOI at a time. Figure 4 depicts the eight steps of the program. We used python to process the steps and to execute SQL queries in the postgres database, whereas steps one to five used postgres and steps five to seven ran in python itself.

Step 1: The program starts by inserting the respective point cloud and EPC data into the database. This reduces the amount of data simultaneously in the database and speeds up GIS-functions such as the intersection function.

Step 2: The program runs batches of footprints iteratively to avoid memory issues. In our experience, a batch size of 500 works well, leading to 120 iterations for an AOI with 60.000 footprints. The program selects all footprints inside the AOI and saves them as a materialized view in the second step. This reduces the computationally expensive intersection of footprints in the AOI at each of the thousands of iterations.

Step 3: To account for small deviations in the spatial correlation between the footprint and the point cloud data, a buffer of 0.5 m is added to each footprint. In Fig. 4, this buffer is indicated by the footprint’s black outlines.

Step 4: Next, all LiDAR points within footprints are selected and assigned to the respective footprint. In Fig. 4 points intersecting with a footprint are plotted in black. Points outside footprints are plotted in orange. In step 4, we also applied a minimum threshold of 100 points to the footprints and filtered out buildings with less points than this threshold. This is indicated by red polygons in Fig. 4. We used this pre-selection to avoid point clouds that did not provide a useful geometric representation of a building and would reduce the quality of the dataset.

Step 5: In step 5, EPC data are matched to the buildings by UPRN. Currently, only a subset of buildings holds EPCs which leads to a significant number of houses without energy attributes (red polygon in Fig. 4). Furthermore, it is possible that one footprint is allocated with multiple EPC if a building contains multiple dwellings. In addition, some footprints do not have a linked UPRN. This fact is discussed in more detail in section Technical Validation.

Step 6: Airborne LiDAR data only covers the buildings’ roofs and fragments of the walls. Therefore, floor points are added to the point cloud in a raster of 0.5 m, because we expected that this improves the representation of the building. The step is conducted in python using the shapely library.

Step 7 and 8: In the end, the point clouds are saved in “.npy” format and the footprint polygons and EPC data are saved in a “.geojson“ file.

## Data Records

The Points for Energy Renovation (PointER) dataset^23^ can be downloaded from 10.14459/2023mp1713501.

### Dataset size and content

The PointER dataset^23^ comprises more than one million point clouds. The selected regions include almost 1.4 million buildings, however our point cloud threshold of 100 points per building point cloud eliminated of around 25% of buildings. As Table 2 shows, this effect was strongest in Plymouth, Coventry, Erewash, and Sutton.

Furthermore, only around 45% of footprints could be linked with EPC data by UPRN. We observed a great variance with 56% in Peterborough and 30% in Sutton. As a result, our final dataset contains around half a million point clouds with energy feature data.

### Dataset structure

Table 3 gives a schematic overview of the dataset’s folder structure. The dataset contains one result folder for each of the 16 selected AOIs, named according to the AOI’s LAD code, e.g. E06000014 for York. Each result folder contains one sub-folder with all building point clouds in python numpy “.npy” format. Building point cloud files are named according to their footprint’s centroid’s coordinate in the spatial reference system EPSG 27700, i.e. “XCOORDINATE_YCOORDINATE.npy”. Point coordinates of a point cloud are in EPSG 27700. In addition, there is a “final_result_AOI_CODE.json” file that maps the building point cloud file to the EPC data. Finally, a summary of the number of footprints with point cloud and EPC information of the AOI is stored in the “production_metrics_AOI_CODE.json” file.

**Table 3:** Folder structure of published dataset.

Result folder	Sub-folder	Point cloud files
E06000014	npy_raw	448435.3287834528_209292.45212838988.npy
		448446.4391428226_209267.82503584144.npy
		…
	final_result_E06000014.json	
	production_metrics_E06000014.json	
E06000026	…	
…	…	
E09000033	…	

Table 4 visualizes the structure of the “final_result_.json” files. The first four columns originate from the point cloud generation process. They connect the footprint with the resulting point cloud file. The information about number of points refers to the building point cloud before adding floor points. This can be used to filter out footprints with too little or too many points depending on the use case. Furthermore, the UPRN column enables linking data to the point cloud.

**Table 4:** Structure of feature table provided in final_result.json.

Data type	Column name	*Description*
**Point cloud data**	id_fp	*Unique footprint identifier*
	pc_file_name	*Filename of point cloud*
	num_p_in_pc	*Number of points in point cloud*
	**uprn**	*UPRN of Verisk dataset*
**Linked EPC data**	LMK_KEY	*Unique EPC identifier*
	BUILDING_REFERENCE_NUMBER	*EPC building reference number*
	CURRENT_ENERGY_RATING	*Energy rating and efficiency score*
	POTENTIAL_ENERGY_RATING	
	CURRENT_ENERGY_EFFICIENCY	
	POTENTIAL_ENERGY_EFFICIENCY	
	PROPERTY_TYPE	*80 columns with more detailed building and energy information*
	…	
	**UPRN**	*UPRN of EPC dataset*
	UPRN_SOURCE	

In the provided dataset, we combined point clouds with EPC data. The EPC features consist of a unique identifier for each EPC entry, current and potential energy rating, current and potential energy efficiency as well as 80 more features with detailed building and energy information. Point cloud data and EPC were linked through the Verisk footprint UPRN and the EPC UPRN.

## Technical Validation

### Quality of input data

The National LiDAR Programme covers the entire area of England. We downloaded the LiDAR point cloud data with resolution of 1 m which translates to an average point cloud density of 1 point per square meter. The National LiDAR Programme offers a metadata dashboard with details on each survey’s mission dates and quality metrics^41^. The average ground truth error across 1215 surveys is 3.66 cm. Furthermore, 97.1% of LiDAR points from overlapping flightlines are less than 15 cm different in elevation^41^.

Verisk’s UKBuildings edition 13 online version dataset includes 28,733,631 buildings from the whole of Great Britain as well as the Belfast urban area. Verisk conducts an internal data quality analysis on accuracy and completeness, but only plans to publish the results in future releases. Therefore, we conducted a qualitative assessment by visualizing the footprint polygons and LiDAR data on Google aerial as depicted in Fig. 2.

Information about the quality of EPC data can be found in the publication’s technical notes^42^. The EPC dataset contains around 60% of the housing stock in England and just less than 60% in Wales^42^. The proportion is similar across all regions in England^42^. The energy assessment of individual buildings is conducted by energy assessors, who are responsible for the robustness of the data in relation to individual buildings. In addition, there are validation checks as the data is uploaded on the registery^42^. With regard to subsequent potential applications, it is important to note that users need to carefully interpret the EPC data. The creation process in the UK has several shortcomings leading to an over-prediction of energy use especially for lower efficiency classes, as described by Few *et al*.^43^.

Finally, Local Authority District boundaries, Output Area boundaries and Rural Urban Classification affect the selection of buildings for point cloud generation, but not the building point clouds itself.

### Quality assessment of resulting building point clouds

**Fig. 5:**
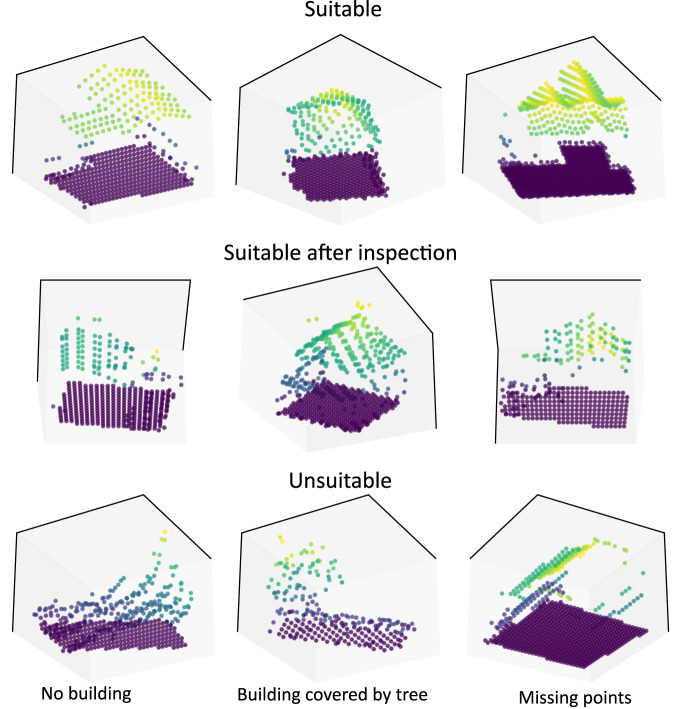
Visualization of building point cloud examples of three assessment categories. Around 85% of building point clouds are “suitable”, 10% are “suitable after inspection” and 5% are “unsuitable”.

Our approach uses building outlines to crop the point clouds. Hence, errors can arise from spatial or temporal mismatch between point cloud data and building footprints. Therefore, we conducted a manual inspection of the resulting building point clouds. To this end, we randomly selected a subset of 5000 point clouds, assuming that the quality of the subset can be extrapolated to the entire dataset. First, we classified the point clouds into “suitable”, “inspection required” and “unsuitable” based on a 2D visualization. In a second round, we inspected the 3D representation as well as an aerial image of the “inspection required” buildings and classified them into “suitable after inspection” and “unsuitable”. Figure 5 gives examples for building point clouds of different quality.

Around 5% of the point clouds were of low-quality, meaning, that the building was not recognizable. There were a number of reasons for this. Some buildings missed points on the roof, usually caused by surrounding structures. Other buildings were covered by vegetation which results in a chaotic point structure. Most of these buildings were small buildings such as garages or auxiliary buildings. Furthermore, a poor spatial alignment between LiDAR data and building outline could led to missing roof structures. The different temporal representations in the two datasets led to some point clouds that appeared to be vegetation or a construction site instead of a building. Finally, for some of the geographic areas, LiDAR points of two surveys overlapped, which could result in distorted building point clouds in a few cases.

After reassessing “inspection required” point clouds in detail, 478 out of 650 buildings were classified as “suitable after inspection”. The vast number of these point clouds were divided into two cases. First, there were buildings that contained a small number of vegetation points covering the roof, but the overall building was clearly recognizable to the human eye. Second, the building point clouds appeared slightly odd at first, but the buildings were part of narrow townhouse-complexes. Therefore, these 478 point clouds were described as edge cases. They made up around 10% of buildings. The remaining 85% of buildings were classified as suitable.

### Effect of point threshold

**Fig. 6:**
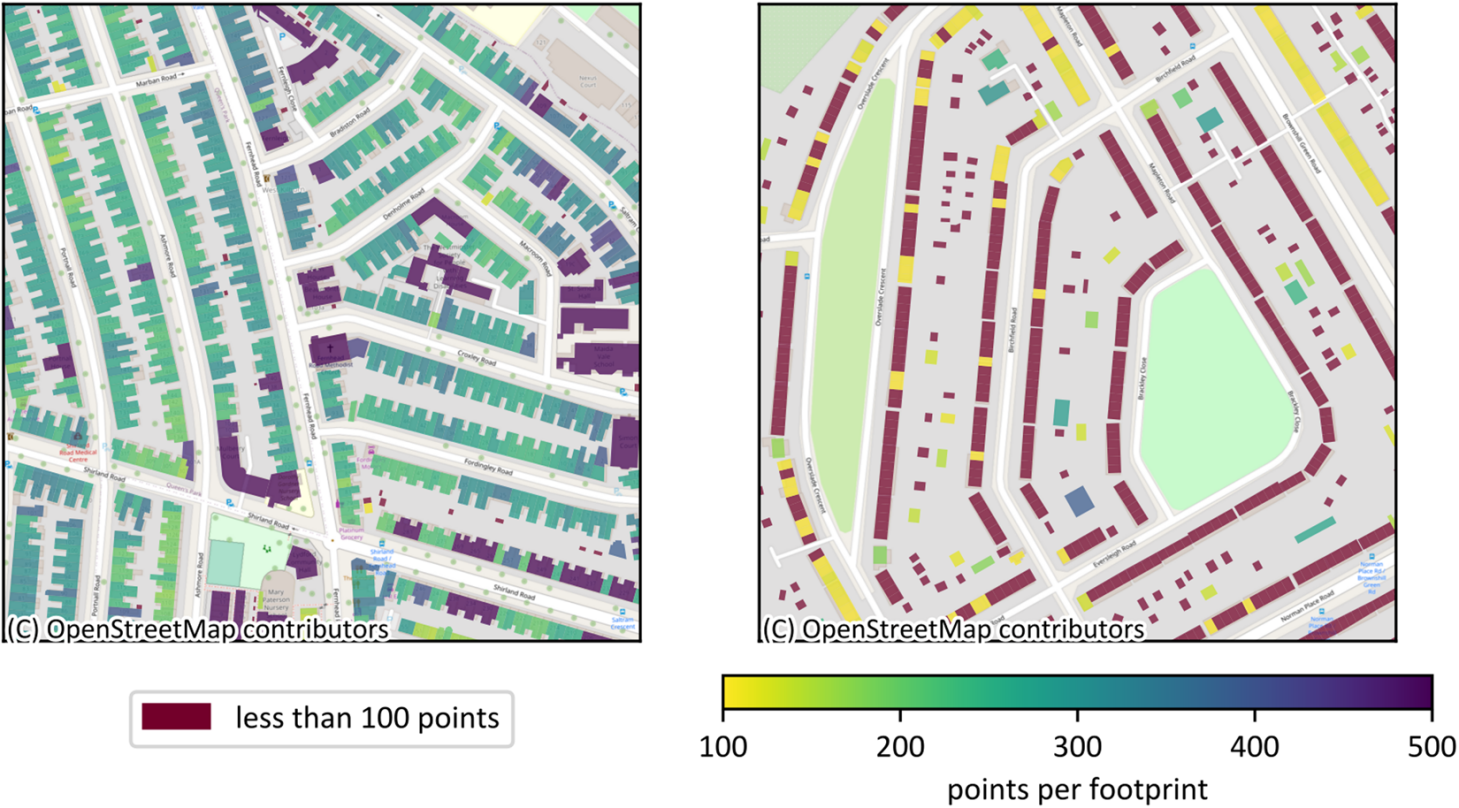
Building footprints of Westminster (left) and Coventry (right) with number of points within their boundary. Westminster has only few buildings with less than 100 points (visualized in red), whereas Coventry displays larger numbers of those footprints in some areas of the city.

Part of our approach is filtering out point clouds that contain less points than a defined threshold of 100 points. This is because buildings with fewer than 100 points are found to lack a rich representation. Figure 6 provides an impression of the number of LiDAR points per footprint for Westminster and Coventry. In Westminster (left) almost all buildings have more points than the threshold, except for a few garage or auxiliary garden buildings, which are visualized in red. On the other hand, the selected district in Coventry includes many buildings with less than 100 points. These are small townhouse buildings that simply have a small footprint area and consequently do not contain enough points. Some of the footprints (yellow) comprise just enough points to be above the threshold. Furthermore, garages and auxiliary buildings, too, are mostly below 100 points. While the point cloud threshold leads to a higher number of well-represented buildings, it also leads to a bias by removing small buildings. This can also be observed in the representativity analysis illustrated in Fig. 8.

### Linkage of EPC features to point clouds through UPRN

**Fig. 7:**
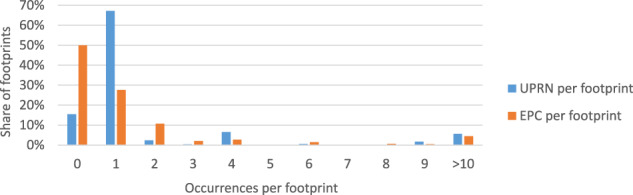
Number of UPRN and EPC data points per footprint for our dataset.

To join the point cloud with EPC data we used the footprint dataset’s UPRN feature. As an alternative, UPRN can be allocated to footprints through spatial intersection. However, the spatial intersection approach led to a lower number of buildings with UPRN. Therefore, we decided to use the UPRN feature provided by Verisk. Figure 7 shows the number of UPRN and EPC entries per footprint for buildings in our dataset.

In our PointER dataset^23^, 1,040,425 or 84.54% of the footprints have at least one UPRN. Furthermore, some footprints are linked to more than one UPRN. As the UPRN refers to a dwelling not to a building, this can be the case for multi-dwelling units. Furthermore, multiple UPRN per footprint could also arise due to erroneous UPRN allocation in some cases. Overall, we linked 27.14% of footprints with exactly one EPC entry and 56.91% of footprints remained without EPC data. Moreover, 15.95% of footprints had multiple EPC values. In our dataset, in the final result data frame, this is reflected by multiple rows that have identical point cloud filenames, but different EPC data. Multiple EPC links require an EPC selection approach. For example, the selection of the average or lowest EPC rating linked to a footprint could be used.

### Comparing england’s building feature distribution with our dataset’s distribution

**Fig. 8:**
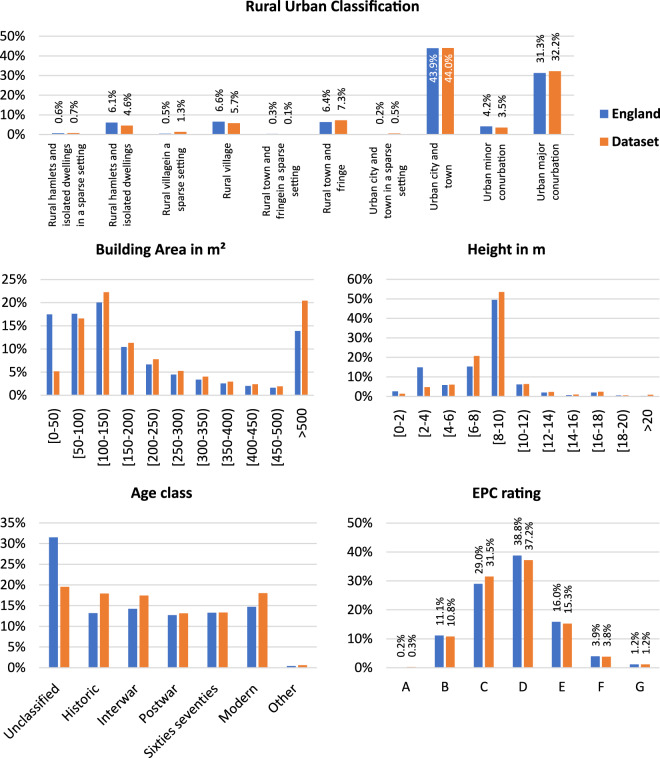
Distribution of Rural Urban Classification, building area, height, age class and EPC rating of our dataset in comparison to all buildings in England included in UK Buildings dataset.

This section evaluates our subset’s representativity in terms of RUC, area, height, age class and EPC rating. The distribution in our dataset in comparison to England is visualized in Fig. 8.

The graph at the top shows the asymmetry between the 10 rural-urban classes, which led to the challenge of achieving exact alignment of the RUC distribution. Nevertheless, the overall representation is similar, especially for the largest classes “Urban city and town”, “Urban major conurbation”, “Rural town and fringe”, “Rural village” and “Rural hamlets and isolated dwellings”. The building characteristics area and height are shown in the two center graphs and display high agreement, except for small buildings with areas under 50 m² and a height of 2–4 m. This can be attributed to our approach of filtering out buildings that contain too few points. Furthermore, the figure depicts a difference in the age class distribution, where our dataset comprises less unclassified buildings than the England dataset. Instead, our dataset contains slightly more modern, interwar, and historic buildings. Finally, a similar distribution of EPC ratings is key for the application in building energy modeling. The bar graph on the bottom right in Fig. 8 indicates a similar distribution of EPC ratings of our dataset in comparison to all of England’s footprints. Therefore, we conclude, that with our approach of selecting building footprints based on RUC, we derive a subset of buildings that possess a similar feature composition as the entire English building stock.

## Usage Notes

As mentioned in the paragraph on UPRN and EPC per footprint, some footprints are linked to multiple energy characteristics, because a building can contain multiple dwellings. When users require linking point clouds to unique energy features, they first need to apply a selection process. As there are multiple approaches with advantages and disadvantages depending on the use case, we leave this step to future users.

Depending on the application, the dataset might need to be normalized (e.g. in a point cloud deep learning pipeline). Currently, coordinates are in the metric coordinate reference system EPSG 27700, the Ordnance Survey National Grid reference system. Buildings can be normalized in relation to the largest building if scale preservation is required. However, most point clouds will consequently only occupy a fraction of the normalized space, because large buildings are less common, as visible in the height and area distribution displayed in Fig. 8.

All data is licensed under the Open Government License, except for the building footprints. Hence, users can exploit the dataset both commercially and non-commercially, but have to acknowledge the sources of the data. The UKBuildings footprint data is provided by Verisk under a private license for research purposes. Therefore, we can use building footprints to generate point clouds, but we cannot include them in this dataset for download. To extend the dataset to other regions, we recommend contacting Verisk for their UKBuildings dataset. Alternatively, other footprint data, such as OSM can be used. When using OSM, regions with high data quality and coverage should be selected.

This dataset can be used in a range of applications. For example, explorative studies can investigate the explanatory power of LiDAR data for building energy characteristics, e.g. through the application of deep learning methods. Recent advances in deep learning methods for point clouds and their application are promising^44–47^ and we expect building point clouds to include significant information. Hence, studies can build point cloud classification models and predict EPC labels for all buildings in England and the UK.

Using our open source code, building point clouds can be generated for any location with available building footprint and LiDAR data. This way, future studies can be conducted across multiple countries. In addition, our dataset could be coupled with additional data sources such as aerial images, street view images, historic energy usage, or socio-demographic data.

Although our dataset is motivated by the challenge of modeling building energy efficiency, it is also relevant for applications outside of this field. Building point clouds can be used to evaluate architectural features or to support urban planning activities. Through the standardized Unique Property Reference Number (UPRN)^30^ other datasets can easily be linked to the building point clouds.

## Data Availability

The code used for generating building point clouds is available at https://github.com/kdmayer/PointER. The repository includes a detailed description of software and python packages used, as well as their versions.
